# Cortical hypertrophies in cementless short stem total hip arthroplasty in young patients under 60 years and elderly patients over 75 years—analysis of outcome and risk factors for cortical hypertrophies

**DOI:** 10.1007/s00402-026-06450-w

**Published:** 2026-09-07

**Authors:** Paul Michael Schwarz, Samuel Stützle, Christian Stadler, Matthias Holzbauer, Lorenz Pisecky, Tobias Gotterbarm, Matthias Luger

**Affiliations:** 1 Department of Orthopaedics and Traumatology, Kepler Universitätsklinikum, Linz, Austria; 2 Johannes Kepler University of Linz, Linz, Austria

**Keywords:** Cortical Hyperthrophies, Short-stem total hip arthroplasty, Fitmore stem, Bone remodeling

## Abstract

**Background:**

Cortical hypertrophy (CH) is a common radiological finding associated with short-stem total hip arthroplasty (THA), but its clinical significance remains unclear. This study evaluates differences in outcomes between young patients under 60 years of age and elderly patients over 75 years of age who underwent cementless short stem THA. The aim was to evaluate if there are differences in these patients depending on the presence of CH.

**Methods:**

A retrospective analysis of 208 short-stem THAs performed between 2014 and 2017 was conducted. Patients were divided into younger (< 60 years, *n* = 119) and older (> 75 years, *n* = 89) groups. Clinical outcomes, including Harris Hip Scores (HHS) and Oxford Hip Scores (OHS), were compared between patients with and without CH. Multivariate regression analysis was performed to identify risk factors for CH, focusing on delta hip offset, canal fill index, and stem alignment.

**Results:**

Younger patients with CH demonstrated significantly better HHS (96.1 ± 7 vs. 90.8 ± 14.3, *p* = 0.010) and OHS (45.8 ± 5.2 vs. 42.9 ± 8.8, *p* = 0.028) compared to those without CH. In older patients, the presence of CH did not significantly impact outcomes. Regression analysis identified younger age (*p* = 0.012), higher Canal Fill Index III (*p* = 0.047), and stem alignment (*p* = 0.049) as significant predictors of CH.

**Conclusion:**

Cortical hypertrophy is associated with non-inferior functional outcomes in younger patients but shows limited clinical relevance in older patients. Implant positioning and patients demographics significantly influence the development of CH. These findings support the interpretation of CH as an adaptive response in younger, active patients rather than a pathological condition.

## Background

Conventional cementless femoral stems show satisfactory clinical and radiographic outcomes over extended follow-up periods [1–4]. The trend towards minimally invasive hip arthroplasty and the goal of reducing bone loss have led to the increasing development and availability of cementless short stems [5]. These types of stems offer several advantages, including preservation of proximal femoral bone stock, less invasive implantation techniques, and the potential for easier revision surgeries [6–8]. As a result, short-stem implants have gained popularity, particularly among younger, active patients who are expected to undergo future revisions [9].

Despite several theoretical advantages, some short stems exhibit radiological alterations, such as cortical hypertrophy (CH). The Fitmore® short stem (Zimmer, Warsaw, IN) is known for its high prevalence of cortical hypertrophy, a radiological finding characterized by localized thickening of the cortical bone. Multiple studies have reported CH associated with this short-stem design, particularly in Gruen zones 3 and 5 [10, 11]. Maier et al. [12] found CH in 63% of patients at early follow-up but noted no adverse impact on clinical outcomes​. Thalmann et al. [13] reported an even higher CH prevalence of 71% at five years, confirming that CH did not negatively affect pain levels or functional scores. However, Innmann et al. [10] observed persistent CH in 56% of patients 6 to 10 years after surgery, raising questions about its long-term biomechanical implications. A recent study of Luger et al. [14] found that CH occurred significantly more frequently in younger patients treated with the Fitmore® stem compared to older patients. This implies that increased mechanical loading and bone remodeling activity may affect CH development in younger adults. Despite these findings, no clear link has been established between CH and complications such as implant loosening or pain, suggesting that it might represent a physiological adaptation rather than a pathological condition, this seems especially true for younger more active patients.

While previous studies have examined the prevalence and clinical impact of CH, the influence of age on CH development remains underexplored. Younger patients, due to higher activity levels and mechanical loading, appear more prone to CH, whereas older patients may have reduced remodeling capacity, potentially affecting clinical and radiological outcomes. This study specifically investigates the role of age in CH development and its clinical implications in short-stem THA. It compares outcomes between younger (< 60 years) and older (> 75 years) patients and seeks to identify risk factors, such as delta hip offset, canal fill index, and stem alignment. These findings aim to address whether CH represents a physiological adaptation or has clinical relevance, forming the basis for our research question.

## Materials and methods

### Study design

This analysis is based on the same institutional registry that formed the basis of a previously published work which compared outcomes of cementless short-stem total hip arthroplasty (THA) in patients younger than 60 and older than 75 years [15]. All patients were treated with total hip arthroplasty (THA) using the same implant combination between 2014 and 2017. In every case, a cementless curved short stem (Fitmore® stem, ZimmerBiomet, Warsaw, IN, USA) was implanted in combination with a cementless titanium press-fit cup with or without screws (Allofit®/-S, ZimmerBiomet, Warsaw, IN, USA) through a minimally invasive anterolateral approach. The study was approved by the institutional review board (protocol number 1239/2019) and conducted in accordance with the 2008 Declaration of Helsinki. Informed consent was obtained from all participants.

The Fitmore® hip stem was first introduced at the authors’ institution in 2011. The first 57 cases performed between 2011 and 2013 were excluded from the study, as they fell within the learning curve period and were often implanted with a different cup system. To ensure sufficient follow-up, only patients who underwent surgery between 2014 and 2017 were included. A further 108 cases were excluded due to incomplete radiological follow-up. From this cohort 208 cases with complete clinical and radiological follow up data were included for final analysis. Patients were categorized into two age groups: younger (< 60 years) and older (> 75 years). The study compared clinical and radiological outcomes, as well as complication and revision rates, between these groups to assess the influence of age on cortical hypertrophy and implant performance.

**Fig. 1:**
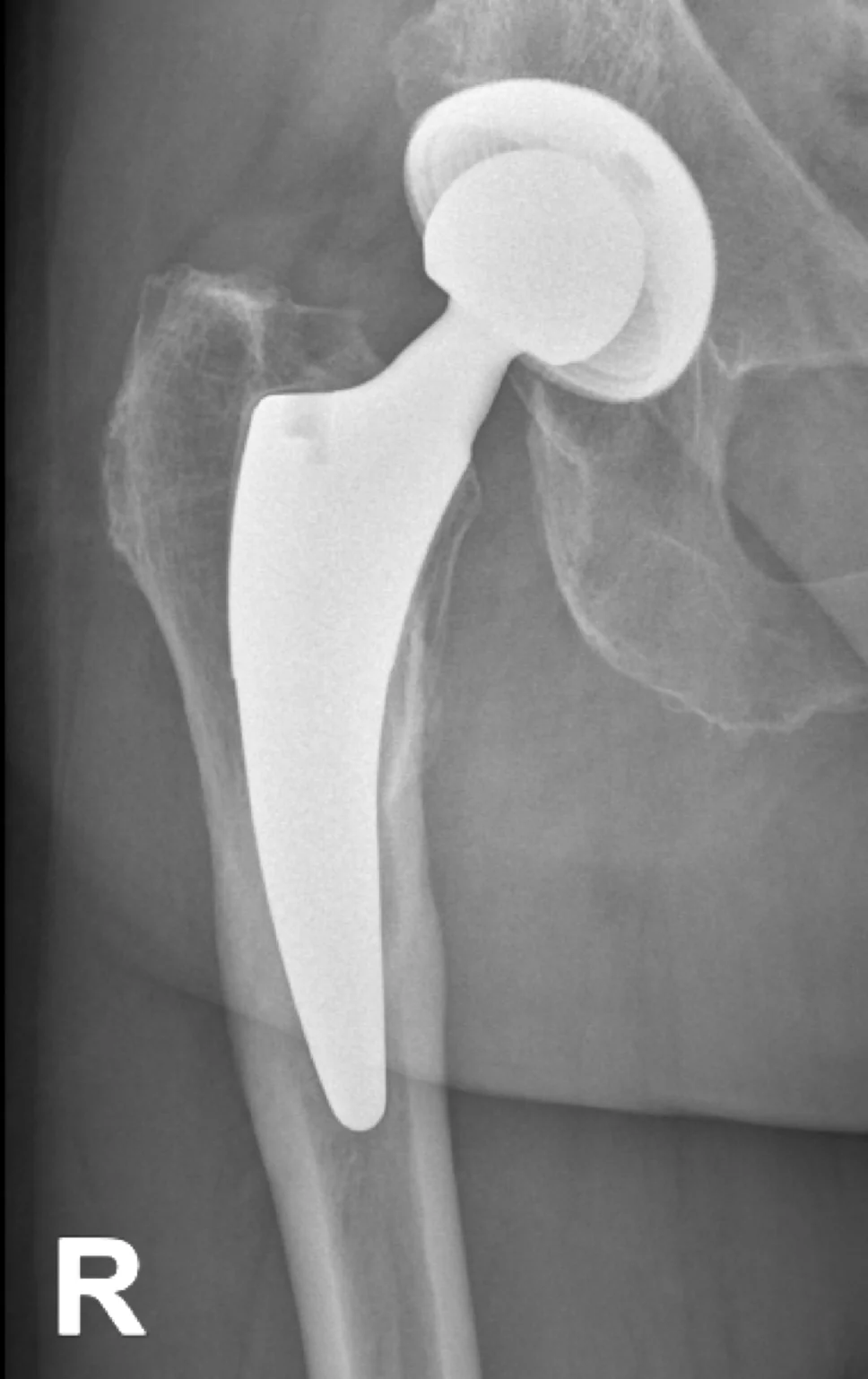
Radiographs of a 67 year old female patient with a short femoral Fitmore stem. Cortical hyperthrophies in Gruen Zone 3, 5 and 6 can be observed

This study aimed to further investigate published data on a cohort which showed a significantly lower numbers of CHs in patients older than 75 years of age compared to control group with patients under 60 years of age at index surgery in short stem THA with Fitmore hip stem. Primary objective was to evaluate, if statistically significant differences in occurrence of CHs in both age groups had any effect on the clinical outcome. The secondary objective was to evaluate any risk factors for occurrence of CHs in the study cohort. Functional outcomes were assessed using validated scoring systems, including the Harris Hip Score (HHS), Oxford Hip Score (OHS), and Forgotten Joint Score (FJS-12). Radiological assessments included the parameters as cortical hypertrophy, radiolucent lines (RL), bone resorption and osteolysis analyzed for the separate Gruen zones [16]. Risk factors for the analysis included sex, age, Body Mass Index (BMI), height, weight, Dorr Type [17], delta hip offset, stem alignment, Canal Fill Index I, Canal Fill Index II, Canal Fill Index III. All radiographic measurements were performed for the present study on calibrated anteroposterior and axial radiographs of the hip using mediCAD® software (Hectec GmbH, Altdorf, Germany). The Canal Fill Index (CFI) was defined as the ratio between the width of the stem and the width of the endosteal femoral canal, measured at three predefined levels: immediately proximal to the lesser trochanter (CFI I), at the level of the lesser trochanter (CFI II), and 2 cm distal to the lesser trochanter (CFI III). Stem alignment was defined as the angle between the longitudinal axis of the stem and the anatomical axis of the femur on the anteroposterior radiograph; a deviation > 3° was classified as varus or valgus alignment, while alignment within ± 3° was considered neutral (see Fig. 1).

### Cohort

**Fig. 2:**
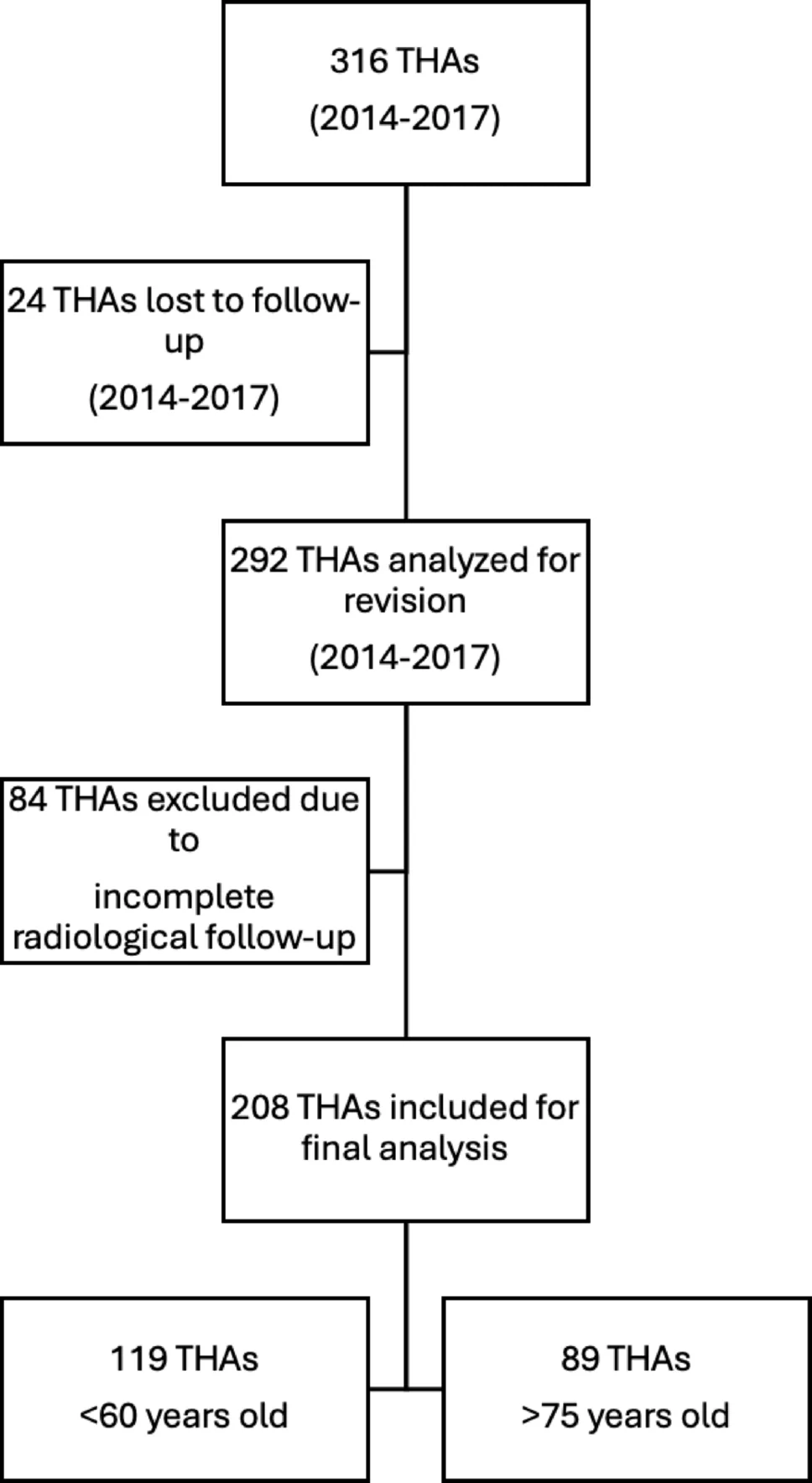
Flow diagram of patient inclusion and exclusion

A cohort of 316 short stem THAs in 300 patients was analyzed for inclusion. To avoid confusion regarding the different sample sizes, the cohort flow is summarized as follows: of the 316 THAs initially screened, 24 were lost to follow-up, leaving 292 cases that were available for the analysis of overall complications and revision rates. Of these 292 cases, a further 84 were excluded due to incomplete radiological follow-up, resulting in 208 THAs with complete clinical and radiological follow-up that formed the basis of the radiological and functional outcome analysis (119 in the younger and 89 in the elderly group). The detailed inclusion and exclusion process is illustrated in the flow diagram (Fig. 2). Two age groups were included for the analysis of clinical and radiological follow-up, as well as of complications and rate of revision. One age group with patients under 60 years of age at index surgery and one group of patients older than 75 years of age at index surgery were included in the study. The younger patients were included as the control group and the elderly patients were included as the treatment group. In the younger control group 149 THAs in 140 patients and in the elderly treatment group 167 THAs in 160 patients were performed between 2014 and 2017. In this cohort 24 cases were lost to follow-up. 292 cases were analyzed for overall revision rates. Due to incomplete radiological follow-up 84 THAs were excluded for final evaluation. Therefore, a cohort comprised of 208 THAs with complete follow up was analyzed. Two age groups were included for the analysis of clinical and radiological follow-up, as well as of complications and rate of revision. One age group of patients under 60 years of age at index surgery and one group of patients older than 75 years of age at index surgery were included in the study. The younger patients were included as the control group and the elderly patients were included as the treatment group. In the younger control group 119 THAs and in the elderly treatment group 89 THAs were performed between 2014 and 2017. Indication for surgery were primary osteoarthritis, avascular necrosis of the femoral head, congenital dysplasia of the hip (Crowe grade I), secondary osteoarthritis such as posttraumatic conditions as well as rheumatoid arthritis. Patients with bilateral THA or prior hip surgery have also been included.

All surgeries were performed at a single tertiary university hospital. Patients were followed at the recommended follow-up examinations. A minimum follow-up of 2 years was required for inclusion. The routine interval for a follow-up examination at the study center are at 3 months, 1, 3, 5, 7 and 10 years postoperatively. If a patient missed a follow-up examination, the patient was invited for a clinical and radiological follow-up examination. If the patient refused to participate personally, the patient was asked for any complication, revision surgery or reoperation within the last follow-up examination given the informed consent. If a patient was deceased, any complication, revision surgery or reoperation was excluded using information from relatives, general practitioners and clinical records. Therefore, both study groups were reviewed in one collective for complications and revisions as well as one collective for clinical and radiological outcome.

### Implants

A cementless, curved short stem (Fitmore® stem, ZimmerBiomet, Warsaw, IN, USA) and a cementless titanium press-fit cup with or without screws (Allofit®/-S, ZimmerBiomet, Warsaw, IN, USA) was implanted in every case. Fitmore® hip stem is a titanium alloy stem (Ti Al6V4) that has a porolock Ti-VPS coating in the proximal part to enhance bone ingrowth and is available in four different neck angle options (127°, 129°, 137°, 140°). A highly cross-linked polyethylene liner (Alpha Durasul®, ZimmerBiomet, Warsaw, IN, USA) and a ceramic femoral head (BIOLOX forte, CeramTec GmbH, DE; Sulox, ZimmerBiomet, Warsaw, IN, USA) were used. Digital templating was conducted in every case using mediCAD® version 5.1 (Hectec GmbH, Altdorf, Germany).

### Surgical technique

In total, 13 surgeons performed the surgeries. The surgeries were performed by 6 consultants and 4 residents. 3 surgeons performed the surgeries as residents and after finishing residency also as consultants. Consultants performed at least 50 arthroplasties per year at the institution. Surgeries performed by residents were always conducted under supervision of a consultant.

All surgeries were performed under laminar air flow. Extremity preparation was performed with threefold antiseptic scrub with alcohol disinfectant in all cases. The standardized peri- and postoperative protocol was identical in all cases, including single-shot antibiotics [cefuroxime 1.5 g intravenous (i.v.), directly preoperatively], full weight-bearing as tolerated from the first postoperative day on, indomethacin 75 mg twice daily for the prevention of heterotopic ossification on days 1–4 postoperatively, and 40 mg low-molecular-weight heparin or rivaroxaban 10 mg for 28 days postoperatively as venous thromboembolic event prophylaxis. Suturing was done either by intracutaneous suturing. Fluoroscopy was not routinely used.

A minimally invasive anterolateral approach was performed in supine positioning [18]. A skin incision was centered over the greater trochanter. An incision at the border between the Tensor fasciae latae and the Tractus iliotibilias was performed. Then, the Watson-Jones interval between Tensor fasciae latae and Gluteus medius was bluntly dissected. A capsulectomy was performed in each case. The average operation time from skin incision to closure was 78.3 min (min.: 48.1; max.: 200 min).

### Statistical analysis

Descriptive statistics were used to summarize patient demographics and outcomes. Normality of continuous variables was assessed with the Shapiro–Wilk test. As the data was normally distributed comparisons between groups were performed using Student’s t-tests for continuous data and Pearson’s chi-square tests for categorical data. Multivariate logistic regression analysis was employed to identify predictors of CH, including patient demographics and surgery-related parameters. Stem survival was measured for revision for any reason and for stem revision by using a Kaplan–Meier survival analysis with a 95% confidence interval (CI). Results were considered statistically significant at *p* < 0.05. Data analysis was conducted using SPSS version 28 (IBM SPSS Statistics, Chicago, IL).

## Results

Demographic data is displayed in Table 1. Both groups demonstrated differences between both age groups. ASA scores varied significantly, with more younger patients classified as ASA 1 (41.2%) compared to older patients, (*p* < 0.001). Dorr classification differed between groups, with younger patients showing a higher rate of Dorr type A femurs (31.1%), while older patients showed more Dorr type C femurs (19.1%, *p* = 0.049).

**Table 1:** Patient demographics

	Young < 60 years of age	Elderly > 75 years of age	*P* value
Total number of hips	119 (112 patients)	89 (84 patients)	
Average Follow-up (in years)	4.4	4.4	0.933
Min.-Max. (in years)	2.1–7.1	2–7.1	
Gender			0.112
Woman	58	53	
Men	61	36	
Age at Surgery (in years)	51.8 ± 6.4	79.1 ± 3.3	** < 0.001**
Indication for surgery			**0.013**
Primary osteoarthritis	93 (78.2%)	77 (86.5%)	
Avascular necrosis of the femoral head	10 (8.4%)	11 (12.4%)	
Congenital dysplasia of the hip	15 (12.6%)	1 (1.1%)	
Secondary osteoarthritis	1 (0.8%)	0 (0.0%)	
Side			0.643
Left	56 (47.1%)	39 (43.8%)	
Right	63 (52.9%)	50 (56.2%)	
ASA Score			** < 0.001**
1	49 (41.2%)	7 (7.9%)	
2	61 (51.3%)	50 (56.2%)	
3	9 (7.6%)	32 (36%)	
4	0 (0.0%)	0 (0.0%)	
BMI	27.5 ± 5.2	27 ± 3.5	0.360
Surgeon’s experience			0.101
Consultant	86 (72.3%)	73 (82%)	
Resident	33 (27.7%)	16 (18%)	
Dorr Classification			**0.049**
A	37 (31.1%)	15 (16.9%)	
B	67 (56.3%)	57 (64%)	
C	15 (12.6%)	17 (19.1%)	

*ASA Score* American Society of Anesthesiologists Score, *BMI* Body Mass Index, kg/m^2^

Bold letters indicate significant values

Radiological outcomes revealed that CH was significantly more prevalent in younger patients (54.6%) than older patients (31.5%, *p* < 0.001), see Table 2. Localization of CH predominantly occurred in Gruen zones 3 and 5, with younger patients showing significantly higher rates of CH in these zones (*p* < 0.001 and *p* = 0.002, respectively). The incidence of radiolucent lines differed, appearing in 13.4% of younger patients compared to 2.2% of older patients (*p* = 0.004).

**Table 2:** Radiological outcome

	Young < 60 years of age	Elderly > 75 years of age	*P* value
Heterotopic ossifications			0.221
Yes (%)	19 (16%)	9 (10.1%)	
No (%)	100 (84%)	80 (89.9%)	
Brooker-Classification			0.088
0	100 (84%)	80 (89.9%)	
1	16 (13.5%)	7 (7.9%)	
2	3 (2.5%)	0 (0.0%)	
3	0 (0.0%)	2 (2.2%)	
Cortical hypertrophy			** < 0.001**
Yes (%)	65 (54.6%)	28 (31.5%)	
No (%)	54 (45.4%)	61 (68.5%)	
Gruen zone 1	0 (0.0%)	0 (0.0%)	-
Gruen zone 2	0 (0.0%)	0 (0.0%)	-
Gruen zone 3	64 (53.8%)	27 (30.3%)	** < 0.001**
Gruen zone 4	0 (0.0%)	0 (0.0%)	-
Gruen zone 5	37 (31.1%)	11 (12.4%)	**0.002**
Gruen zone 6	0 (0.0%)	0 (0.0%)	-
Gruen zone 7	0 (0.0%)	0 (0.0%)	-
Radiolucent Line (< 2 mm)			**0.004**
Yes (%)	16 (13.4%)	2 (2.2%)	
No (%)	103 (86.6%)	87 (97.8%)	
Gruen zone 1	9 (7.6%)	0 (0.0%)	**0.008**
Gruen zone 2	1 (0.8%)	0 (0.0%)	0.386
Gruen zone 3	3 (2.5%)	0 (0.0%)	0.131
Gruen zone 4	3 (2.5%)	2 (2.2%)	0.898
Gruen zone 5	2 (1.7%)	0 (0.0%)	0.219
Gruen zone 6	0 (0.0%)	0 (0.0%)	-
Gruen zone 7	2 (1.7%)	0 (0.0%)	0.219
Bone resorption			0.658
Yes (%)	7 (5.9%)	4 (4.5%)	
No (%)	112 (94.1%)	85 (95.5%)	
Gruen zone 1	0 (0.0%)	0 (0.0%)	-
Gruen zone 2	0 (0.0%)	0 (0.0%)	-
Gruen zone 3	0 (0.0%)	0 (0.0%)	-
Gruen zone 4	0 (0.0%)	0 (0.0%)	-
Gruen zone 5	0 (0.0%)	0 (0.0%)	-
Gruen zone 6	0 (0.0%)	0 (0.0%)	-
Gruen zone 7	7 (5.9%)	4 (4.5%)	0.658
Osteolysis			-
Yes (%)	0.0 (0%)	0.0 (0%)	
No (%)	100 (100%)	100 (100%)	

Bold letters indicate significant values

Clinical outcomes are displayed in Table 3. Younger patients exhibited higher activity levels and better quality of life compared to older patients. UCLA Activity Scores were significantly higher in younger patients (6.9 ± 2.3 vs. 4.6 ± 2.3, *p* < 0.001). Younger patients also reported better quality of life as measured by EQ-5D-3L scores (82.3 ± 19 vs. 75.9 ± 11.9, *p* = 0.003). Pain relief, as assessed by VAS, was comparable between the groups, with no significant differences (*p* = 0.102).

**Table 3:** Clinical outcome for both groups

	Young < 60 years of age	Elderly > 75 years of age	*P* value
Pain			0.146
Yes (%)	42 (35.3%)	23 (25.8%)	
No (%)	77 (64.7%)	76 (74.2%)	
VAS (± SD)	0.85 ± 1.4	0.56 ± 1.1	0.102
Harris Hip Score (± SD)	93.7 ± 11.2	91.9 ± 8.8	0.224
Oxford Hip Score (± SD)	44.5 ± 7.2	43.7 ± 5.3	0.350
Forgotten Joint Score 12 (± SD)	81.7 ± 26.3	81.5 ± 22	0.952
WOMAC Score (± SD)	7.4 ± 14.8	9.3 ± 12.3	0.334
UCLA Score (± SD)	6.9 ± 2.3	4.6 ± 2.3	** < 0.001**
EQ-5D-3L (%)	82.3 ± 19	75.9 ± 11.9	**0.003**
EQ-5D-3L Q1 (median)	2.4 (1)	1.3 (1)	0.160
EQ-5D-3L Q2 (median)	1.1 (1)	1.2 (1)	** < 0.001**
EQ-5D-3L Q3 (median)	1.1 (1)	1.4 (1)	** < 0.001**
EQ-5D-3L Q4 (median)	1.4 (1)	1.6 (2)	**0.002**
EQ-5D-3L Q5 (median)	1.1 (1)	1.2 (1)	0.075
VR-12 PCS (± SD)	54.7 ± 10.9	47.9 ± 10.2	** < 0.001**
VR-12 MCS (± SD)	43.4 ± 4.3	44.3 ± 5.8	0.238

Bold letters indicate significant values

The presence of CH had a notable impact on functional outcomes in younger patients but not in older ones. Younger patients with CH demonstrated significantly higher Harris Hip Scores (96.1 ± 7 vs. 90.8 ± 14.3, p = 0.010) and Oxford Hip Scores (45.8 ± 5.2 vs. 42.9 ± 8.8, *p* = 0.028) compared to those without CH, see Table 4. In contrast, no significant differences in functional outcomes were observed in older patients with or without CH with Harris Hip Scores of 91.2 ± 10.1 compared to 92.2 ± 8.1 (*p* = 0.614), and Oxford Hip Scores of 42.8 ± 5.5 compared to 44.1 ± 5.3 (*p* = 0.317).

**Table 4:** Clinical outcome depending on the presence of cortical hypertrophies for both age groups

	Presence of CHs	No Presence of CHs	*P* value
Young < 60 years of age			
Pain			**0.007**
Yes (%)	16 (24.6%)	26 (48.1%)	
No (%)	49 (75.4%)	28 (51.9%)	
VAS (± SD)	0.6 ± 1.2	1.2 ± 1.5	**0.013**
Harris Hip Score (± SD)	96.1 ± 7	90.8 ± 14.3	**0.010**
Oxford Hip Score (± SD)	45.8 ± 5.2	42.9 ± 8.8	**0.028**
Forgotten Joint Score 12 (± SD)	87.3 ± 21.4	75 ± 30.1	**0.010**
WOMAC Score (± SD)	5.3 ± 11.5	9.9 ± 17.8	0.093
UCLA Score (± SD)	7 ± 2.3	6.7 ± 2.3	0.389
EQ-5D-3L (%)	83.5 ± 19.1	81 ± 19	0.484
EQ-5D-3L Q1 (median)	3.4 (1)	1.2 (1)	0.237
EQ-5D-3L Q2 (median)	1.1 (1)	1 (1)	0.056
EQ-5D-3L Q3 (median)	1.2 (1)	1.1 (1)	0.120
EQ-5D-3L Q4 (median)	1.5 (1)	1.3 (1)	0.051
EQ-5D-3L Q5 (median)	1.1 (1)	1.1 (1)	0.387
VR-12 PCS (± SD)	44 ± 2.9	42.7 ± 5.5	0.089
VR-12 MCS (± SD)	56.2 ± 9.2	52.8 ± 12.5	0.072
Elderly > 75 years of age			
Pain			0.902
Yes (%)	7 25.0%	16 26.2%	
No (%)	21 75.0%	45 73.8%	
VAS (± SD)	0.6 ± 1.2	0.5 ± 1	
Harris Hip Score (± SD)	91.2 ± 10.1	92.2 ± 8.1	0.614
Oxford Hip Score (± SD)	42.8 ± 5.5	44.1 ± 5.3	0.317
Forgotten Joint Score 12 (± SD)	82.4 ± 18.9	81.1 ± 23.4	0.793
WOMAC Score (± SD)	10.6 ± 14.9	8.6 ± 11	0.480
UCLA Score (± SD)	4.4 ± 2.4	4.7 ± 2.3	0.668
EQ-5D-3L (%)	77.9 ± 12.6	75 ± 11.6	0.296
EQ-5D-3L Q1 (median)	1.4 (1)	1.3 (1)	0.789
EQ-5D-3L Q2 (median)	1.3 (1)	1.3 (1)	0.835
EQ-5D-3L Q3 (median)	1.4 (1)	1.4 (1)	0.519
EQ-5D-3L Q4 (median)	1.6 (1)	1.7 (1)	0.489
EQ-5D-3L Q5 (median)	1.3 (1)	1.2 (1)	0.343
VR-12 PCS (± SD)	47.1 ± 11.8	48.1 ± 9.5	0.655
VR-12 MCS (± SD)	43.7 ± 7.2	44.5 ± 5.1	0.514

Bold letters indicate significant values

Multivariate regression analysis identified several key predictors of CH development. In the general cohort younger age was significantly associated with the presence of CHs (OR 0,970, CI 0.947–0.993; p = 0,012), as well as higher Canal Fill Index III (OR 56,804, CI 1047–3082,839; *p* = 0.047). A varus stem alignment showed a significantly increased OR for the presence of CHs in elderly patients (OR 1204, CI 1001–1.44, *p* = 0.049), see Table 5.

**Table 5:** Multivariate regression analysis of different potential risk factors for the different age groups

	CI (95% Confidence Intervall)	*P* value
Total		
Sex	1.867 (0.804–4.337)	0.146
Age	0.970 (0.947–0.993)	**0.012**
BMI	1.239 (0.664–2.313)	0.501
Height	1.072 (0.878–1.308)	0.498
Weight	0.929 (0.752–1.149)	0.497
Dorr Type	0.879 (0.502–1.537)	0.650
Δ Hip offset	1.042 (0.987–1.101)	0.139
Stem alignment (°)	1.034 (0.919–1.163)	0.580
Canal Fill Index I	2.783 (0.006–1209.257)	0.741
Canal Fill Index II	0.095 (0.000–53.929)	0.467
Canal Fill Index III	56.804 (1.047–3082.839)	**0.047**
Young < 60 years of age		
Sex	2.775 (0.899–8.560)	0.076
BMI	1.744 (0.777–3.918)	0.178
Height	1.186 (0.919–1.532)	0.189
Weight	0.823 (0.628–1.080)	0.160
Dorr Type	0.923 (0.434–1.964)	0.836
Δ Hip offset	1.053 (0.974–1.138)	0.191
Stem alignment (°)	0.901 (0.761–1.068)	0.230
Canal Fill Index I	0.349 (0.000–735.189)	0.787
Canal Fill Index II	0.064 (0.000–249.115)	0.514
Canal Fill Index III	190.575 (0.874–41574.507)	0.056
Elderly > 75 years of age		
Sex	1.043 (0.209–5.193)	0.959
BMI	1.071 (0.734–1.565)	0.721
Height	1.033 (0.920–1.159)	0.585
Weight	0.982 (0.871–1.107)	0.770
Dorr Type	0.750 (0.285–1.974)	0.560
Δ Hip offset	1.031 (0.942–1.127)	0.508
Stem alignment (°)	1.204 (1.001–1.447)	**0.049**
Canal Fill Index I	29.794 (0.000–4069791.733)	0.574
Canal Fill Index II	0.031 (0.000–2653.573)	0.549
Canal Fill Index III	12.845 (0.016–10264.775)	0.454

Bold letters indicate significant values

Revision-free survival and revision rates for the 292 cases regardless of complete radiological follow-up are summarized in Table 6. Mean follow-up was comparable between younger and older patients (4.5 vs. 4.6 years, *p* = 0.571). Revisions for any reason occurred in 3 cases (2.2%) in patients younger than 60 years and in 8 cases (5.2%) in patients older than 75 years, without a statistically significant difference between groups (*p* = 0.169). Stem revisions were required in 2 younger patients (1.4%) and 8 elderly patients (5.2%) (*p* = 0.479). Reoperations without component exchange were rare and observed in one case (0.7%) in the younger group and in none of the elderly patients (*p* = 0.293).

**Table 6:** Revisions and reoperations

	Young < 60 years of age	Elderly > 75 years of age	*P* value
Average Follow-up (in years)	4.5	4.6	0.571
Min.-Max. (in years)	0.05–8	0–8.6	
Revisions	93.7 ± 11.2	91.9 ± 8.8	0.224
Revisions for any reason	3 (2.2%)	8 (5.2%)	0.169
Stem Revisions	2 (1.4%)	8 (5.2%)	0.479
Reoperation	1 (0.7%)	0 (0.0%)	0.293

Bold letters indicate significant values

In the subgroup of 208 cases with complete radiological follow-up, revision rates were additionally analyzed according to the presence of cortical hypertrophies (see Table 7). Revision for any reason occurred in 1 of 115 cases without CH (0.9%) and in 1 of 93 cases with CH (1.1%), without a statistically significant difference between groups (*p* = 0.880). No stem revisions were recorded in this radiologically complete cohort. When stratified by age within this subgroup, revision occurred in 1 of 65 younger cases with CH (1.5%) and in none of the 54 younger cases without CH, while in elderly patients revision was observed in 1 of 61 cases without CH (1.6%) and in none of the 28 cases with CH (*p* = 0.496).

**Table 7:** Revisions and reoperations according to the presence of cortical hypertrophies (208 cases with complete radiological follow-up)

	No CH	CH	*P* value
Total number of hips	115	93	
Revisions for any reason	1 (0.9%)	1 (1.1%)	0.880
Stem Revisions	0 (0.0%)	0 (0.0%)	–
Reoperation	1 (0.9%)	0 (0.0%)	0.367

*CH* Cortical Hypertrophy

Bold letters indicate significant values

## Discussion

The aim of this study was to analyse short-stem total hip arthroplasties (THA) as well as to evaluate clinical and radiological outcomes, with a focus on differences between the younger and older patient cohort, the impact of cortical hypertrophy on function, and the risk factors contributing to cortical hypertrophy development. The primary objective was to assess whether CH influences clinical outcomes, implant behaviour across age groups, and to identify predictors for its occurrence in short-stem implants.

Our findings demonstrated a significantly higher prevalence of CH in younger patients compared to older individuals. The observed differences between the groups could be related to increased mechanical loading and a grater bone remodeling capacity typically observed in younger patients. The expanded analysis further suggests that younger patients who developed CH might achieve better functional outcomes. These observations indicate that CH might represent a beneficial response rather than a complication, especially in younger more active individuals. Specifically, younger patients with CH showed significantly higher Harris Hip Scores and Oxford Hip Scores compared to those without CH. These results indicate that CH may enhance load distribution, reduce micromotion, and contribute to improved implant stability particularly in patients with higher bone remodeling potential and physical activity. This interpretation aligns with findings from Iwase et al. [19], who reported lower rates of thigh pain in patients with CH, further supporting the concept of CH as an adaptive rather than pathological process. In contrast, older patients in our cohort did not experience significant functional differences between the CH and non-CH group. This may reflect reduced physiological bone responsiveness or activity levels in older individuals, where CH plays a less dynamic role in postoperative outcomes. These results suggest that CH may serve as a surrogate marker of healthy bone remodelling in younger, more active adults. It should, however, be emphasized that the better functional scores observed in younger patients with CH should not be interpreted as a direct causal effect of cortical thickening on patient-reported outcomes. A plausible mechanism is that CH and superior PROMs are both downstream expressions of a shared underlying factor rather than one causing the other: higher physical activity and better pre-operative bone quality generate the increased cyclic mechanical loading that stimulates cortical remodeling and the same higher activity level and bone quality are themselves associated with better functional scores [20, 21]. In this interpretation CH acts as a radiological marker of a mechanically active, well-remodeling bone–implant interface rather than as the cause of the improved outcome. Because activity level and pre-operative bone quality were not available as continuous covariates in the present cohort, this potential confounding could not be adjusted for, and the association between CH and functional outcome must therefore be regarded as associative rather than causal.

Our findings fall within the mid-range of CH prevalence reported in the literature. Thalmann et al. [13] and Maier et al. [12] reported higher CH rates of 71% and 63%, respectively, both in younger patient populations undergoing short-stem THA. In contrast, Iwase et al. [19] and Fujita et al. [22] reported a prevalence of 9,5% and 20% after 5 and 10 years respectively, additionally Cho et al. [23] observed only 6.2% at an average follow-up of 54.6 months. Such discrepancies likely stem from differences in implant design, patient age, surgical technique, and biomechanical loading conditions. Freitag et al. [24] conducted a 10-year longitudinal study on Fitmore stems and found a CH prevalence of 26%, primarily located in Gruen zones 3 and 5. They found no adverse impact on functional outcomes, supporting the hypothesis that CH represents a physiological adaptation to implant stress distribution rather than a pathological change. More recently, Maier et al. [12] provided valuable long-term evidence with 10-year prospective data on 80 Fitmore® short stems. They reported persistent CH in 74% of cases mainly in Gruen zones 2, 3, 5 and 6 accompanied by cortical thinning in up to 80% and ongoing stem subsidence averaging 5 mm at 10 years. Despite these pronounced radiological changes, no correlation was found with thigh pain, Harris Hip Score, or Oxford Hip Score, confirming that even progressive CH and subsidence over a decade do not translate into poorer clinical outcomes. This strengthens the interpretation of CH as a stable, long-term adaptive remodeling process rather than a pathological reaction.

Regarding symptoms, most studies indicate that CH is not associated with increased pain. Crawford et al. [25] reported no correlation between distal femoral CH and thigh pain, despite observing CH in 74% of cases in Gruen zone 3 and 56% in zone 5. Similarly, the lower incidence of thigh pain in the CH group indicates a potential stabilizing effect of cortical hypertrophy on the stem, thereby reducing discomfort as described by Iwase et al. [19], Thalmann et al. [13] further reinforced this, finding no significant difference in function or pain in patients with and without CH at 5-year follow-up.

However, a few studies present contrasting evidence. Hallam et al. [26] described a correlation between CH and thigh pain in patients with cemented titanium stems, although their small sample size and unique implant design limit the generalizability of their findings. Likewise, Hayashi et al. [27] reported a significant association between CH in Gruen zone 4 and thigh pain in short tapered wedge stems. Similarly, Kanto et al. [28] observed that diffuse-type CH involving multiple green zones was frequently followed by stress shielding, suggesting that extensive cortical remodelling may in some cases, precede unfavourable load redistribution rather than physiological adaptation. Their results emphasize that the relationship between CH and clinical outcomes like depend on the extent and pattern of CH.

Our multivariate analysis identified several key risk factors for CH. Younger age was a significant predictor, consistent with findings by Cho et al. [23],Ishii et al. [29] as well as Ritter et al. [30] also observed that patients with CH were nearly ten years younger than those without, while Katsimihas et al. reported a smaller age gap of 3.5 years, supporting our finding that younger, more active patients are more prone to cortical remodeling. Interestingly, Ritter et al. [30] reported higher CH prevalence in women, whereas Katsimihas et al. [31] found it more common in men particularly those with Charnley grade A and avascular necrosis. Canal Fill Index (CFI) also emerged as a significant determinant, underscoring the role of metaphyseal stem engagement in stimulating cortical remodeling. Stem alignment was another contributing factor, especially in older patients. Both Iwase et al. [19] and Goto et al. [32] found that varus and valgus stem positioning significantly increased the likelihood of developping CH. Although their findings broadly align with ours, caution is warranted with a direct comparison due to the different stem designs used in their studies.

This study has several limitations. Its retrospective design introduces selection and recall biases. The single center design may limit generalizability of its findings. The fact that this cohort has been previously analysed in earlier publications, may reduce its degree of novelty. Its inclusion criteria focused exclusively on patients younger than 60 and older than 75 years, the exclusion of the intermediate age group is another potential limitation. No randomization was performed, which introduces the possibility of unrecognized confounders influencing the outcomes. In particular, individual physical activity level and pre-operative bone quality were not available as quantitative variables for all patients and could therefore not be entered as covariates in the regression model. As both factors are plausibly associated with the development of cortical hypertrophy as well as with functional outcome, the observed association between CH and higher Harris Hip and Oxford Hip Scores in younger patients may at least in part be explained by such residual confounding rather than by a direct biomechanical effect of cortical hypertrophy itself. This finding should therefore be regarded as hypothesis-generating, and prospective studies adjusting for activity level and bone quality are warranted to confirm whether CH is an independent marker of favourable functional outcome.

## Conclusion

Cortical hypertrophy is associated with non-inferior functional outcomes in younger patients but shows limited clinical relevance in older patients. Implant positioning and patients’ demographics significantly influence the development of CH. These findings support the interpretation of CH as an adaptive response in younger, active patients rather than a pathological condition.

## Data Availability

The datasets analyzed during the current study are not publicly available due to institutional data protection regulations and patient confidentiality requirements but are available from the corresponding author on reasonable request and with permission of the institutional review board.

## References

[1] Hartzband MA, Glassman AH, Goldberg VM, Jordan LR, Crowninshield RD, Fricka KB, Jordan LC (2010) Survivorship of a low-stiffness extensively porous-coated femoral stem at 10 years. Clin Orthop Relat Res 468(2):433–440 19557489 10.1007/s11999-009-0950-3 PMC2807009

[2] Aldinger PR, Jung AW, Breusch SJ, Ewerbeck V, Parsch D (2009) Survival of the cementless Spotorno stem in the second decade. Clin Orthop Relat Res 467(9):2297–2304 19504161 10.1007/s11999-009-0906-7 PMC2866918

[3] Capello WN, D’Antonio JA, Jaffe WL, Geesink RG, Manley MT, Feinberg JR (2006) Hydroxyapatite-coated femoral components: 15-year minimum follow-up. Clin Orthop Relat Res 453:75–80 17016216 10.1097/01.blo.0000246534.44629.b2

[4] De Martino I, De Santis V, D’Apolito R, Sculco PK, Cross MB, Gasparini G (2017) The synergy cementless femoral stem in primary total hip arthroplasty at a minimum follow-up of 15 years. Bone Joint J 99-B(1):29–36 28053254 10.1302/0301-620X.99B1.BJJ-2016-0231.R1

[5] Giardina F, Castagnini F, Stea S, Bordini B, Montalti M, Toni A (2018) Short stems versus conventional stems in cementless total hip arthroplasty: a long-term registry study. J Arthroplasty 33(6):1794–1799 29395723 10.1016/j.arth.2018.01.005

[6] Liang HD, Yang WY, Pan JK, Huang HT, Luo MH, Zeng LF, Liu J (2018) Are short-stem prostheses superior to conventional stem prostheses in primary total hip arthroplasty? A systematic review and meta-analysis of randomised controlled trials. BMJ Open 8(9):e021649 30244208 10.1136/bmjopen-2018-021649 PMC6157567

[7] de Waard S, van der Vis J, Venema PA, Sierevelt IN, Kerkhoffs GM, Haverkamp D (2021) Short-term success of proximal bone stock preservation in short hip stems: a systematic review of the literature. EFORT Open Rev 6(11):1040–1051 34909223 10.1302/2058-5241.6.210030 PMC8631238

[8] Corradi N, Trimarchi A, Manca F, Martini I, Colombelli A, Belluati A (2026) The use of short cementless femoral stems in total hip arthroplasty for femoral neck fractures: a scoping review of the literature. Musculoskelet Surg 110(2):139–147 40782324 10.1007/s12306-025-00917-6

[9] Thorey F, Hoefer C, Abdi-Tabari N, Lerch M, Budde S, Windhagen H (2013) Clinical results of the Metha short hip stem: a perspective for younger patients? Orthop Rev (Pavia) 5(4):e34 24416478 10.4081/or.2013.e34 PMC3883075

[10] Innmann MM, Weishorn J, Bruckner T, Streit MR, Walker T, Gotterbarm T, Merle C, Maier MW (2019) Fifty-six percent of proximal femoral cortical hypertrophies 6 to 10 years after total hip arthroplasty with a short cementless curved hip stem—a cause for concern? BMC Musculoskelet Disord 20(1):261 31142303 10.1186/s12891-019-2645-6 PMC6542080

[11] Maier KS, Schiener T, Frigg A (2025) Progression of cortical hypertrophy, subsidence and thigh pain after short curved Fitmore femoral hip stem over 10 years. J Orthop Surg Res 20(1):649 40660277 10.1186/s13018-025-06064-9 PMC12257828

[12] Maier MW, Streit MR, Innmann MM, Krüger M, Nadorf J, Kretzer JP, Ewerbeck V, Gotterbarm T (2015) Cortical hypertrophy with a short, curved uncemented hip stem does not have any clinical impact during early follow-up. BMC Musculoskelet Disord 16:371 26627999 10.1186/s12891-015-0830-9 PMC4667403

[13] Thalmann C, Kempter P, Stoffel K, Ziswiler T, Frigg A (2019) Prospective 5-year study with 96 short curved Fitmore hip stems shows a high incidence of cortical hypertrophy with no clinical relevance. J Orthop Surg Res 14(1):156 31133027 10.1186/s13018-019-1174-1 PMC6537407

[14] Luger M, Holzbauer M, Klotz MC, Fellner F, Gotterbarm T (2024) Cementless short stem total hip arthroplasty in patients older than 75 years: is it feasible? Arch Orthop Trauma Surg 144(8):3715–3727 38967777 10.1007/s00402-024-05425-z PMC11417050

[15] Luger M (2023) A comparison of outcome in cementless short stem total hip arthroplasty in patients under 60 and over 75 years. Dissertation, Johannes Kepler University Linz. Published as: Luger M, Holzbauer M, Klotz MC, Fellner F, Gotterbarm T (2024) Cementless short stem total hip arthroplasty in patients older than 75 years: is it feasible? Arch Orthop Trauma Surg 144(8):3715–3727 10.1007/s00402-024-05425-z PMC11417050 38967777

[16] Gruen TA, McNeice GM, Amstutz HC (1979) “Modes of failure” of cemented stem-type femoral components: a radiographic analysis of loosening. Clin Orthop Relat Res 141:17–27 477100

[17] Dorr LD, Faugere MC, Mackel AM, Gruen TA, Bognar B, Malluche HH (1993) Structural and cellular assessment of bone quality of proximal femur. Bone 14(3):231–242 8363862 10.1016/8756-3282(93)90146-2

[18] Chen D, Berger RA (2013) Outpatient minimally invasive total hip arthroplasty via a modified Watson-Jones approach: technique and results. Instr Course Lect 62:229–236 23395028

[19] Iwase T, Morita D, Takemoto G (2020) The effects of patient characteristics and stem alignment on distal femoral cortical hypertrophy after cemented polished tapered stem implantation. Eur J Orthop Surg Traumatol 30(4):559–567 31853636 10.1007/s00590-019-02605-1

[20] Pearson OM, Lieberman DE (2004) The aging of Wolff’s “law”: ontogeny and responses to mechanical loading in cortical bone. Am J Phys Anthropol 125(Suppl 39):63–99 10.1002/ajpa.20155 15605390

[21] Kawano T, Nankaku M, Murao M, Goto K, Kuroda Y, Kawai T et al (2022) Development of a clinical prediction rule to identify physical activity after total hip arthroplasty. Arch Phys Med Rehabil 103(10):1975–1982 35421394 10.1016/j.apmr.2022.03.015

[22] Fujita H, Katayama N, Iwase T, Otsuka H (2012) Multi-center study of use of the Exeter stem in Japan: evaluation of 1000 primary THA. J Orthop Sci 17(4):370–376 22552547 10.1007/s00776-012-0237-5

[23] Cho YJ, Chun YS, Rhyu KH, Baek JH, Liang H (2016) Distal femoral cortical hypertrophy after hip arthroplasty using a cementless double-tapered femoral stem. J Orthop Surg (Hong Kong) 24(3):317–322 28031498 10.1177/1602400309

[24] Freitag T, Fuchs M, Friedrich D, Bieger R, Reichel H, Oltmanns M (2024) The migration pattern of a short-tapered femoral stem correlates with the occurrence of cortical hypertrophies: a 10-year longitudinal study using Ein Bild Röntgen Analyse-Femoral Component Analysis. J Clin Med 13(12):3616 38930145 10.3390/jcm13123616 PMC11205188

[25] Crawford DA, Adams JB, Morris MJ, Berend KR, Lombardi AV Jr (2021) Distal femoral cortical hypertrophy not associated with thigh pain using a short stem femoral implant. Hip Int 31(6):722–728 32186204 10.1177/1120700020913872

[26] Hallam P, Haddad F, Cobb J (2004) Pain in the well-fixed, aseptic titanium hip replacement: the role of corrosion. J Bone Joint Surg Br 86(1):27–30 14765860

[27] Hayashi S, Hashimoto S, Matsumoto T, Takayama K, Niikura T, Kuroda R (2020) Risk factors of thigh pain following total hip arthroplasty with short, tapered-wedge stem. Int Orthop 44(12):2553–2558 32767085 10.1007/s00264-020-04762-z

[28] Kanto M, Fukunishi S, Fukui T, Nishio S, Fujihara Y, Okahisa S, Takeda Y, Yoshiya S, Tachibana T (2020) Radiological evaluation of the relationship between cortical hypertrophy and stress shielding after total hip arthroplasty using a cementless stem. Arthroplast Today 6(4):894–900 33204784 10.1016/j.artd.2020.09.018 PMC7649111

[29] Ishii S, Homma Y, Baba T, Shirogane Y, Kaneko K, Ishijima M (2021) Does increased diameter of metal femoral head associated with highly cross-linked polyethylene augment stress on the femoral stem and cortical hypertrophy? Int Orthop 45(5):1169–1177 33619587 10.1007/s00264-021-04994-7

[30] Ritter MA, Fechtman RW (1988) Distal cortical hypertrophy following total hip arthroplasty. J Arthroplasty 3(2):117–121 3397741 10.1016/s0883-5403(88)80076-7

[31] Katsimihas M, Katsimihas G, Lee MB, Learmonth ID (2006) Distal femoral cortical hypertrophy: predisposing factors and their effect on clinical outcome. Hip Int 16(1):18–22 19219773 10.5301/hip.2008.967

[32] Goto K, Furuya Y, Oda K, Minami R, Sano K, Sugimoto M, Matsuda S (2018) Long-term results of total hip arthroplasty using Charnley Elite-Plus stem and the effect of stem geometry on radiographic distal femoral cortical hypertrophy. J Orthop Sci 23(2):365–370 29276040 10.1016/j.jos.2017.12.003

